# Texture analysis of multiparametric magnetic resonance imaging for differentiating clinically significant prostate cancer in the peripheral zone

**DOI:** 10.55730/1300-0144.5633

**Published:** 2023-02-01

**Authors:** Halil ÖZER, Mustafa KOPLAY, Ahmet BAYTOK, Nusret SEHER, Lütfi Saltuk DEMİR, Abidin KILINÇER, Mehmet KAYNAR, Serdar GÖKTAŞ

**Affiliations:** 1 Department of Radiology, Faculty of Medicine, Selcuk University, Konya, Turkey; 2 Department of Public Health, Faculty of Medicine, Necmettin Erbakan University, Konya, Turkey; 3 Department of Urology, Faculty of Medicine, Selcuk University, Konya, Turkey

**Keywords:** Prostate cancer, texture analysis, magnetic resonance imaging, radiomics

## Abstract

**Background/aim:**

Texture analysis (TA) provides additional tissue heterogeneity data that may assist in differentiating peripheral zone (PZ) lesions in multiparametric magnetic resonance imaging (mpMRI). This study investigates the role of magnetic resonance imaging texture analysis (MRTA) in detecting clinically significant prostate cancer (csPCa) in the PZ.

**Materials and methods:**

This retrospective study included 80 consecutive patients who had an mpMRI and a prostate biopsy for suspected prostate cancer. Two radiologists in consensus interpreted mpMRI and performed texture analysis based on their histopathology. The first-, second-, and higher-order texture parameters were extracted from mpMRI and were compared between groups. Univariate and multivariate logistic regression analyses were performed using the texture parameters to determine the independent predictors of csPCa. Receiver operating characteristic (ROC) curve analysis was conducted to assess the diagnostic performance of the texture parameters.

**Results:**

In the periferal zone, 39 men had csPCa, while 41 had benign lesions or clinically insignificant prostate cancer (cisPCa). The majority of texture parameters showed statistically significant differences between the groups. Univariate ROC analysis showed that the ADC mean and ADC median were the best variables in differentiating csPCa (p < 0.001). The first-order logistic regression model (mean + entropy) based on the ADC maps had a higher AUC value (0.996; 95% CI: 0.989–1) than other texture-based logistic regression models (p < 0.001).

**Conclusion:**

MRTA is useful in differentiating csPCa from other lesions in the PZ. Consequently, the first-order multivariate regression model based on ADC maps had the highest diagnostic performance in differentiating csPCa.

## 1. Introduction

Prostate cancer is the second most frequently diagnosed cancer in men and the fifth leading cause of death worldwide [1,2]. Diagnosing prostate cancer consists of digital rectal examination, assessing serum prostate-specific antigen (PSA) levels, and transrectal ultrasound (US)-guided prostate biopsy. However, there are some limitations to these methods. Serum PSA level has a low specificity (25%–40%) and can lead to unnecessary biopsies. Transrectal US-guided biopsy does not usually allow direct visualization of abnormal regions and focuses on the peripheral zone (PZ), missing clinically significant prostate cancer (csPCa) and overdiagnosing csPCa. The detection of csPCa and the evaluation of their biological aggressiveness are important for treatment [3–5].

Multiparametric magnetic resonance imaging (mpMRI) of the prostate is useful in detecting csPCa [5–9]. csPCa has been defined on histology as Gleason scores of ≥7 (including 3 + 4 with a prominent but not predominant Gleason 4 component), volume of ≥ 0.5 mL, or extra prostatic extension [8]. mpMRI based on the second version of the Prostate Imaging Reporting and Data System (PI-RADS) shows high sensitivity and moderate specificity for the detection of csPCa. In a metaanalysis including 21 studies, the diagnostic sensitivity and specificity of PI-RADS v2 were 89% and 73%, respectively, in detecting csPCa [6]. The limitations of mpMRI are the differences in imaging quality between centers and the differences in interpretation between readers [9–12]. Various strategies such as the combination of laboratory tests and imaging and quantitative analysis of images are still being investigated to further improve the diagnostic accuracy of mpMRI in detecting csPCa [13–18].

Texture analysis (TA) is a quantitative image analysis method that can evaluate the signal heterogeneity of the images by examining the distribution and connectivity of pixel intensities within normal and pathological tissues. In medical images, macroscopic heterogeneity can reflect histopathological heterogeneity [19–22]. Recently, in studies on breast, brain, and rectal cancers, magnetic resonance imaging texture analysis (MRTA) has been used for cancer detection, staging, and treatment response assessment. These studies have demonstrated that TA can be a powerful noninvasive tool for the assessment of intratumoral features and thus can be used in evaluating malignancies [19–25].

TA provides additional tissue heterogeneity data that may assist in differentiating PZ lesions in mpMRI. This study investigates the role of MRTA in detecting csPCa in patients with suspicion of prostate cancer.

## 2. Materials and methods

### 2.1. Study design

Between January 2018 and January 2020, 127 patients who had a clinical suspicion of prostate cancer and underwent mpMRI were evaluated retrospectively. mpMRI scans were accessed via the picture archiving and communication system. The following inclusion criteria were queried from our electronic hospital medical records system: (1) mpMRI performed within 6 months before prostate biopsy; (2) csPCa in the PZ using targeted biopsy or radical prostatectomy; and (3) benign lesions or clinically insignificant prostate cancer (cisPCa) in the PZ using targeted or systematic biopsy. Patients with csPCa in the transition zone (n = 10), those who did not have mpMRI (n = 13), those who did not have pathological diagnosis (n = 6), those who had imaging artifacts (n = 6), those who had previous treatment (n = 7), and those who had biopsy before mpMRI (n = 5) were excluded from the study. Finally, 80 consecutive cases were included in the study. The study was approved by the institutional ethics committee of our hospital, and the requirement for written informed consent was waived.

### 2.2. Imaging protocol

MRI examinations were performed using a 3T scanner (MAGNETOM Skyra; Siemens Healthineers AG, Erlangen, Germany) with a pelvic-phased array coil. The MRI protocols were consistent with PI-RADS v2.1. All patients received a spasmolytic to reduce bowel peristalsis. The MRI protocols included axial, coronal, and sagittal T2-weighted turbo spin-echo sequence (TR/TE, 5000/110; echo-train length, 23; number of signals averaged, 3; FOV, 200 × 200 mm), diffusion-weighted imaging (DWI) with a single-shot echo planar imaging sequence (TR/TE, 4500/76; flip angle, 90°; slice thickness, 3.5 mm; matrix size, 128 × 128; FOV, 200 × 200 mm; b values, 50, 1000, and 1500 s/mm^2^), and dynamic contrast-enhanced MRI (DCE-MRI) with a T1-weighted volumetric interpolated breath-hold examination sequences (TR/TE, 5.08/1.77; flip angle 15°; FOV, 259 × 259 mm; slice thickness, 3.5 mm without inter slice gap; temporal resolution, 8 seconds; 35 contrast-enhanced phases acquired sequentially). On dynamic series, after the first two phases, 0.1 mmol/kg of gadobutrol (Gadovist; Bayer Schering Pharma GmbH, Berlin, Germany) was injected intravenously at a rate of 3 mL/s with an MR-compatible automatic injector, followed by 30 mL of saline flush.

### 2.3. Biopsy technique

mpMRI-directed transrectal US-guided fusion biopsy was performed for the targeted lesions. Suspected lesions in mpMRI were localized on the sector map before fusion biopsy. At least three biopsies were performed for the targeted lesions. In patients without suspicious lesions, transrectal US-guided systematic biopsy was performed. All biopsy cores were evaluated histopathologically. Gleason scores of 3 + 4 or more and the maximum cancer core length involvement of 4 mm or more were defined as csPCa [26].

### 2.4. Textural analysis

TA allows quantitative assessment of the heterogeneity of the tumors by analyzing the distribution and relationship of pixel intensities on the medical images. Different methods of TA exist such as statistical, structural, model-based, and transformation-based. The statistical method has been most widely used in TA and includes first-, second-, and higher-order statistics [21,22,27,28].

First-order statistics, also called histogram analysis, evaluate frequency distribution of pixel intensity values within a region of interest (ROI). These forms of textural analysis consider only pixel intensity and do not provide spatial information between pixels. The most common histogram features include mean, standard deviation (SD) or variance, skewness (asymmetry), kurtosis (pointedness or flatness), first-order entropy (irregularity), and mean of positive pixels [21,22,27,28].

More complex computations such as second- or higher-order statistics, which explore the relationship between pixels within the ROI, provide spatial information between pixels. Second-order statistics, such as second-order entropy, energy, homogeneity, dissimilarity, and correlation, are based on gray-level cooccurrence matrix (GLCM) and compare the relationship between two pixels. GLCM is a two-dimensional histogram capturing the frequency of cooccurrence of pixel pairs of certain values in a given spatial range. Higher-order statistics are based on neighborhood gray-level difference matrix (NGLDM) and include contrast, coarseness, and busyness. NGLDM can demonstrate coarseness and complexity within an image by comparing the relationship between more than two pixels [21,22,27,28].

### 2.5. Image analysis

**Figure 1:**
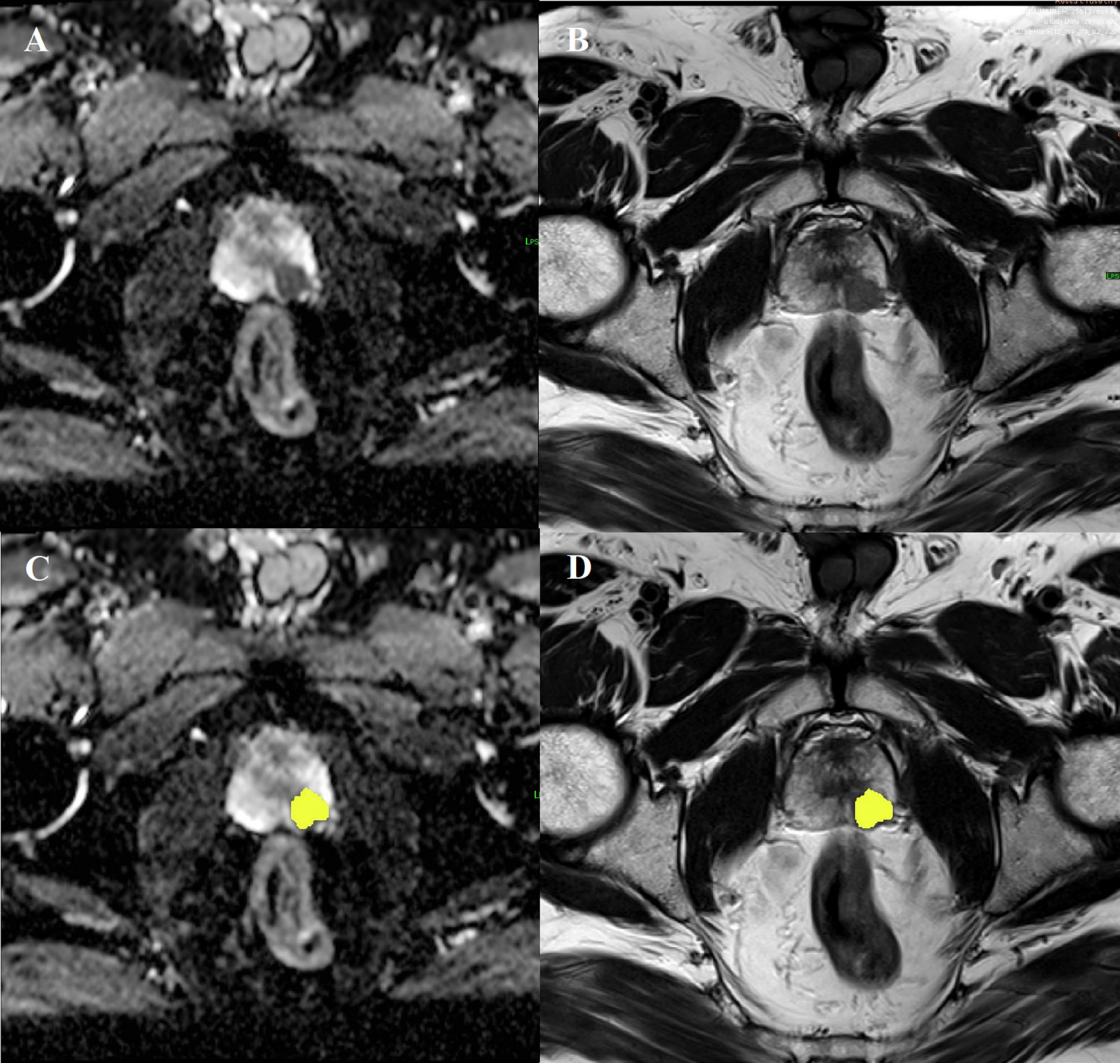
68-year-old man with prostate cancer. (a) ADC map and (b) T2WI shows csPCa located in the left peripheral zone in the apex of the prostate and a region of interest placed (yellow area) on the (c) ADC map and (d) T2WI.

Two radiologists (6 years and 3 years of experience, respectively, in mpMRI interpretation), aware of all clinical, and histopathological findings, interpreted mpMRI in consensus. According to the histopathological findings, csPCa and benign lesions or cisPCa were identified at dedicated workstations. All images were imported to commercially available software (Olea Sphere^®^ 3.0, Olea Medical, La Ciotat, France) for segmentation and TA. The ROI was drawn using the freehand technique to encompass the entire lesion on the ADC map, T2-weighted images (T2WIs), and early and late postcontrast T1-weighted images (T1WIs) in patients with csPCa. In patients with benign lesions or cisPCa, the ROI was determined to include the lesion if visible on mpMRI, and to include the entire peripheral zone if it is not visible or has indistinct margins. After the segmentation, the first-, second-, and higher-order texture parameters were extracted from the selected ROI (Figures 1a–1d). The texture features included first-order parameters (mean, median, skewness, kurtosis, entropy, and uniformity), GLCM (contrast, correlation, difference entropy, joint energy, joint entropy, sum entropy, and inverse variance), and NGLDM (contrast, coarseness, complexity, busyness, and strength).

### 2.6. Statistical analysis

The data were analyzed using SPSS v. 17.0 (IBM Corporation, Armonk, NY, USA) and MedCalc for Windows, version 19.2.6 (MedCalc Software Ltd., Mariakerke, Belgium). The normality of the distribution of continuous numerical variables was analyzed using the Shapiro–Wilk test. Descriptive statistics were presented as mean ± SD or median (range) for continuous numerical variables and count (percentage) for categorical variables.

The Student *t*-test was used to compare two independent groups. Univariate and multivariate logistic regression analyses were performed using texture parameters to define the independent predictors of csPCa. The variables highly associated with each other were excluded from the regression model due to a multicollinearity problem using the backward stepwise analysis method used in multivariate regression analysis. Odds ratios (ORs) were used to denote the effect size of the variables in the regression model. The diagnostic performance of univariate and multivariate texture parameters was assessed using receiver operating characteristic (ROC) curve analysis. During multivariate analysis, the area under curve (AUC) values were derived according to the predictive values of the regression model. Comparisons among AUC values were assessed using the Delong method [29]. In all statistical analyses in this study, p-values of <0.05 were used to denote statistical significance.

## 3. Result

The study enrolled 80 patients (males) with a mean age of 68.05 ± 8.45 years (range, 50–87 years). For all patients, the mean prostate volume was 61.85 ± 34.43 mL and the mean PSA level was 9.76 ± 8.70 ng/mL. Histopathological analysis showed that 39 patients (48.8%) had csPCa, while 41 patients (51.2%) had benign lesions or cisPCa.

Tables 1 and 2 summarize the mean and SD of the texture parameters in groups with csPCa and benign lesions or cisPCa.

### 3.1. Texture features: ADC maps

Except the kurtosis, all first-order texture parameters in csPCa in the PZ were significantly different. In csPCa; mean, median, and entropy were significantly lower, while skewness and uniformity were significantly higher (p < 0.01).

Except the GLCM correlation, GLCM difference entropy, NGLDM coarseness, NGLDM complexity, and NGLDM strength, all second- and higher-order texture parameters were significantly different in csPCa in the PZ. Furthermore, csPCa had significantly lower GLCM joint entropy, GLCM inverse variance, and GLCM sum entropy while having significantly higher GLCM contrast, GLCM joint energy, NGLDM contrast, and NGLDM busyness (p < 0.05).

### 3.2. Texture features: T2WIs

All first-order texture parameters in csPCa in PZ showed significant differences; mean, median, and entropy were lower, while skewness, kurtosis, and uniformity were higher (p < 0.05).Except the GLCM difference entropy, NGLDM coarseness, NGLDM complexity, and NGLDM strength, all second- and higher-order texture parameters in csPCa in the PZ were significantly different. Furthermore, GLCM correlation, GLCM joint entropy, GLCM inverse variance, and GLCM sum entropy were significantly lower for csPCa, whereas GLCM contrast, GLCM joint energy, NGLDM contrast, and NGLDM busyness were significantly higher (p < 0.05).

### 3.3. Texture features: T1WIs

On early postcontrast T1WIs, entropy was significantly lower among the first-order texture parameters, whereas uniformity was significantly higher in csPCa in PZ (p < 0.05).

On late postcontrast T1WIs, mean, median, and uniformity were significantly higher among the first-order texture parameters, whereas skewness and entropy were significantly lower in csPCa in PZ (p < 0.05).

On early and late postcontrast T1WIs, GLCM difference entropy, GLCM joint entropy, and GLCM sum entropy were significantly lower in csPCa in PZ, whereas GLCM contrast, GLCM joint energy, and NGLDM busyness were significantly higher. Furthermore, GLCM correlation in csPCa in the PZ was significantly lower on late postcontrast T1WIs (p < 0.05).

On early and late postcontrast T1WIs, there was no significant difference in other texture parameters in the PZ (p > 0.05).

### 3.4. Logistic regression analysis of texture parameters

The results of the univariate and multivariate logistic regression analyses on texture parameters are shown in Tables 3 and 4.

Among the first-order texture parameters, mean and entropy and, among the second- and higher-order texture parameters, GLCM contrast, GLCM difference entropy, GLCM joint energy, GLCM joint entropy, GLCM sum entropy, and NGLDM busyness were independent predictors of csPCa on the ADC maps.

### 3.5. ROC analysis of the texture parameters and regression models

**Figure 2:**
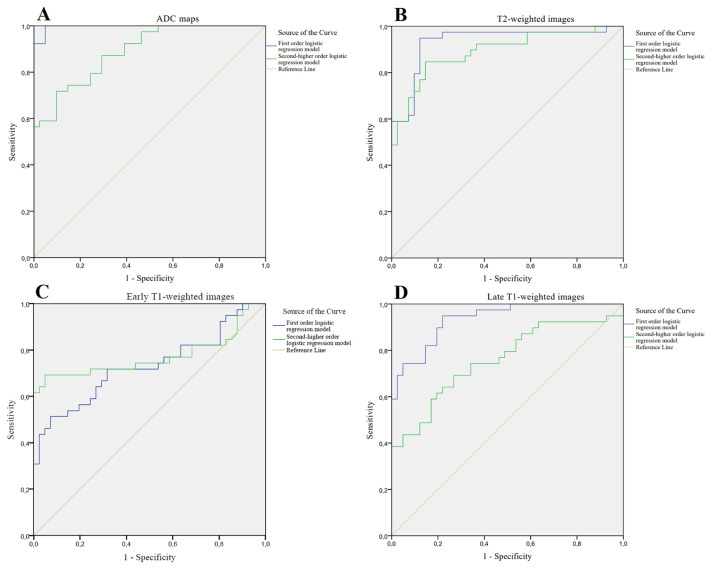
Receiver operating characteristic (ROC) curves of the multivariate logistic regression models based on (a) ADC maps, (b) T2-weighted images, (c) early and (d) late T1-weighted images for the diagnosis of csPCa in the peripheral zone.

The ROC analysis results of multivariate logistic regression models for detecting csPCa in PZ are shown in Table 5. The ROC curves constructed from output probabilities of logistic regression models are shown in Figures 2a–2d.

Univariate ROC analysis showed that the ADC mean and ADC median had the highest AUC value (0.986; 95% CI, 0.969–1 and 0.987; 95% CI, 0.971–1, respectively) for differentiating csPCa in PZ (p < 0.001). In addition, the first-order entropy based on the ADC maps had a higher AUC value (0.920; 95% CI, 0.865–0.975) than other texture features (p < 0.001).

The first-order logistic regression model (mean + entropy) based on the ADC maps had a higher AUC value (0.996; 95% CI, 0.989–1) than other texture-based logistic regression models (p < 0.001).

## 4. Discussion

Recently, radiomics has been used for cancer detection, staging, and treatment response assessment [13,30,31]. Moreover, radiomics has been used in mpMRI, which is widely used for prostate cancer detection, staging, and treatment response. This study showed that texture features derived from mpMRI can be used to distinguish csPCa in PZ. Therefore, we conclude that TA provides additional tissue heterogeneity data that may contribute in differentiating PZ lesions on mpMRI.

TA assesses tumor heterogeneity by analyzing the distribution and relationship of pixel intensities in medical images and provides quantitative information about the lesion that cannot be distinguished by a radiologist [19–22,28].

Studies have shown that TA could be useful in diagnosing prostate cancer [19–22,28,31,32]. In a study on transrectal US, TA could distinguish prostate cancer from benign lesions with a sensitivity of 86% and a specificity of 88% [32]. In addition, a few studies have applied TA on mpMRI [19–22]. Wibmer et al. stated that second-order MRTA is useful in prostate cancer detection and Gleason score assessment [21]. Sidhu et al. showed that MRTA of the prostate transition zone may discriminate csPCa [20]. Niu et al. reported that tumor aggressiveness in prostate cancer can be assessed with specific parameters delivered from MRTA [19]. In our study, we also showed that MRTA can offer additives to distinguish csPCa in PZ.

The mean ADC values vary according to the cell membranes, nuclei, and extracellular space at the tissue level. The mean ADC values are low in high-cellular tumors [33]. Studies have shown that ADC is an appropriate biomarker for diagnosing prostate cancer and its aggressiveness [16,18]. In this study, univariate ROC analysis showed that the ADC mean and ADC median were the best variables in differentiating csPCa in PZ. In addition, the first-order multivariate regression model obtained by adding entropy to the ADC mean had the highest diagnostic performance in differentiating csPCa in PZ. However, the ADC mean values can vary according to the pulse sequences in different devices and different *b* values, which limit the role of the ADC mean values in the distinction between benign and malignant lesions and the cut-off value [33].

In this study, various first-order (skewness, entropy, and uniformity), GLCM (correlation, difference entropy, joint energy, sum entropy) and NGLDM (busyness) texture parameters provided good diagnostic performance for identifying csPCa. Among these texture parameters, first-order entropy obtained from the ADC maps had the excellent diagnostic performance and the higher AUC value. In addition, other entropy parameters obtained using second-order statistics showed significant diagnostic performance. Entropy indicates uncertainty/randomness within the image. High entropy values represent an increase in heterogeneity in the image [22,28,31]. We showed that csPCa lesions were more homogeneous with lower entropy values than benign lesions or cisPCa. Although this seems incompatible with other studies, it is compatible with the definitions of PI-RADS scores [8,16,21]. PI-RADS 4–5 lesions appeared focal, circumscribed, moderately homogeneous, or markedly hypointense focus/mass. PI-RADS 2–3 lesions, on the other hand, were linear or wedge-shaped, and had an indistinct margin and variable signal intensity within the normal PZ [8].

Uniformity, which is inversely correlated with entropy, is a measure of the gray-level distribution of the image; lower values represent heterogeneity [22,28,31]. In this study, high uniformity values were obtained, which showed that csPCa lesions were more homogeneous relative to entropy. In addition, csPCa had high GLCM joint energy values, another parameter showing homogeneity among second-order parameters.

Skewness measures the asymmetry of the histogram curve and can be negative or positive [22,28,31]. We found positive skewness values in csPCa. However, conflicting results in the skewness values have been reported in several studies on prostate cancer [18–20,34]. Its mechanism is unclear, and what it represents is unknown.

Contrast, also called variance or inertia, represents local signal intensity variation in an image; higher values indicate a greater difference in signal intensity between adjacent voxels. Correlation measures the linear dependency of signal intensity between voxel pairs. It can be positive or negative and takes values between 0 (uncorrelated) and 1 (perfectly correlated) [22,28,31]. We found higher GLCM contrast values and lower GLCM correlation values in csPCa, which conforms to the literature [19,21,35]. Busyness is a measure of the intensity change between pixels and their neighborhood [28]. In this study, NGLDM busyness values were higher in csPCa, and no data were found in PubMed for the performance of NGLDM texture parameters in the diagnosis of prostate cancer at the time of writing.

Multivariate regression analysis showed that models based on ADC maps had higher diagnostic performance in the assessment of PZ lesions than T2WI and DCE-MRI. Texture parameters derived from ADC maps have provided more diagnostic benefits in the diagnosis of prostate cancer [22]. In the PI-RADS v2 and v2.1, it was emphasized that DWI should be the basis of scoring lesions in the PZ, and our results are consistent with the PI-RADS [8,36]. First-order texture parameters are better understood and have more clinical validity than second- and higher-order texture parameters [22]. In this study, better diagnostic performance was obtained in models derived from first-order parameters.

Some limitations in this study should be considered. First, the sample size is small. Second, the PI-RADS score of the lesions was not considered in the study. Prospective TA studies using the PI-RADS in a larger series will form the basis of machine learning techniques for prostate cancer. Finally, mpMRI-directed transrectal US-guided fusion biopsy was used as a reference standard instead of radical prostatectomy in most patients.

In conclusion, MRTA is useful in differentiating csPCa from benign lesions or cisPCa. In addition, the first-order multivariate regression model obtained by adding entropy to the mean had the highest diagnostic performance in identifying csPCa.

## Figures and Tables

**Table 1:** Texture parameters of clinically significant prostate cancer and benign lesions or clinically insignificant prostate cancer extracted from ADC maps and T2-weighted images in the peripheral zone.

	ADC maps			T2-weighted images		
	Benign lesions or cisPCa	csPCa	p †	Benign lesions or cisPCa	csPCa	p †
***First-order***						
*Mean*	1404.64 ± 218.43	721.40 ± 170.92	**<0.001**	511.17 ± 197.98	256.57 ± 120.31	**<0.001**
*Median*	1432.85 ± 235.43	690.56 ± 194.62	**<0.001**	513.30 ± 201.29	241.18 ± 85.62	**<0.001**
*Skewness*	−0.489 ± 0.565	0.433 ± 0.681	**<0.001**	−0.113 ± 0.545	0.309 ± 0.488	**<0.001**
*Kurtosis*	3.34 ± 1.19	3.29 ± 1.09	0.827	3.05 ± 0.61	3.56 ± 1.15	**0.019**
*Entropy*	5.29 ± 0.25	4.57 ± 0.51	**<0.001**	5.40 ± 0.15	4.98 ± 0.38	**<0.001**
*Uniformity*	0.031 ± 0.007	0.042 ± 0.021	**0.004**	0.028 ± 0.003	0.038 ± 0.011	**<0.001**
***Second-higher-order***						
*GLCM contrast*	74.33 ± 54.94	151.26 ± 158.41	**0.006**	84.74 ± 28.21	153.28 ± 119.20	**0.001**
*GLCM correlation*	0.728 ± 0.209	0.623 ± 0.306	0.076	0.662 ± 0.128	0.507 ± 0.174	**<0.001**
*GLCM difference entropy*	3.76 ± 0.29	3.61 ± 0.55	0.131	4.12 ± 0.20	4.20 ± 0.42	0.291
*GLCM joint energy*	0.006 ± 0.004	0.019 ± 0.028	**0.007**	0.0032 ± 0.0008	0.0077 ± 0.0086	**0.003**
*GLCM joint entropy*	7.65 ± 0.76	6.87 ± 1.69	**0.011**	8.50 ± 0.37	7.76 ± 1.26	**0.001**
*GLCM invers variance*	0.177 ± 0.053	0.144 ± 0.071	**0.026**	0.144 ± 0.022	0.124 ± 0.044	**0.011**
*GLCM sum entropy*	5.71 ± 0.54	5.10 ± 1.20	**0.006**	6.01 ± 0.21	5.46 ± 0.60	**<0.001**
*NGLDM coarseness*	0.072 ± 0.024	0.075 ± 0.037	0.624	0.029 ± 0.010	0.036 ± 0.024	0.104
*NGLDM contrast*	0.62 ± 0.75	8.17 ± 17.32	**0.010**	0.45 ± 0.14	3.00 ± 7.43	**0.039**
*NGLDM business*	0.009 ± 0.005	0.024 ± 0.015	**<0.001**	0.023 ± 0.008	0.041 ± 0.040	**0.009**
*NGLDM complexity*	3459.83 ± 1258.17	3548.75 ± 1385.14	0.764	5475.10 ± 1110.99	5673.03 ± 1774.22	0.549
*NGLDM strength*	89.96 ± 46.26	97.31 ± 52.62	0.509	34.93 ± 13.11	42.00 ± 28.04	0.158

† Student’s t-test, mean ± standard deviation;

cisPCa, clinically insignificant prostate cancer; csPCa, clinically significant prostate cancer; ADC, apparent diffusion coefficient; GLCM, gray-level cooccurrence matrix; NGLDM, neighborhood gray-level different matrix.

**Table 2:** Texture parameters of clinically significant prostate cancer and benign lesions or clinically insignificant prostate cancer extracted from early and late T1-weighted images in the peripheral zone.

	Early T1-weighted images			Late T1-weighted images		
	Benign lesions or cisPCa	csPCa	p †	Benign lesions or cisPCa	csPCa	p †
***First-order***						
*Mean*	153.37 ± 235.42	171.69 ± 95.15	0.653	92.04 ± 40.41	161.16 ± 80.03	**<0.001**
*Median*	154.20 ± 241.92	169.15 ± 88.59	0.717	91.50 ± 40.45	161.69 ± 80.03	**<0.001**
*Skewness*	0.148 ± 0.555	0.145 ± 0.671	0.982	0.199 ± 0.566	−0.083 ± 0.627	**0.037**
*Kurtosis*	2.89 ± 0.69	3.029 ± 1.360	0.570	2.98 ± 0.96	3.07 ± 0.94	0.667
*Entropy*	5.09 ± 0.30	4.54 ± 0.89	**0.001**	5.00 ± 0.32	4.50 ± 0.79	**0.001**
*Uniformity*	0.035 ± 0.007	0.076 ± 0.097	**0.011**	0.037 ± 0.009	0.057 ± 0.036	**0.002**
***Second-higher-order***						
*GLCM contrast*	163.27 ± 87.97	306.47 ± 404.75	**0.036**	157.99 ± 75.82	259.57 ± 237.18	**0.014**
*GLCM correlation*	0.548 ± 0.197	0.464 ± 0.295	0.135	0.57 ± 0.19	0.40 ± 0.32	**0.005**
*GLCM difference entropy*	4.12 ± 0.20	3.63 ± 0.85	**0.001**	4.08 ± 0.19	3.58 ± 0.85	**0.001**
*GLCM joint energy*	0.010 ± 0.003	0.037 ± 0.062	**0.010**	0.010 ± 0.003	0.036 ± 0.058	**0.008**
*GLCM joint entropy*	6.78 ± 0.49	6.00 ± 1.77	**0.012**	6.79 ± 0.52	5.98 ± 1.76	**0.008**
*GLCM invers variance*	0.110 ± 0.029	0.092 ± 0.052	0.074	0.102 ± 0.037	0.091 ± 0.054	0.328
*GLCM sum entropy*	5.24 ± 0.34	4.52 ± 1.37	**0.003**	5.26 ± 0.35	4.43 ± 1.38	**0.001**
*NGLDM coarseness*	0.063 ± 0.024	0.069 ± 0.076	0.671	0.063 ± 0.027	0.060 ± 0.028	0.645
*NGLDM contrast*	1.88 ± 1.45	139.64 ± 598.99	0.159	2.24 ± 2.04	65.87 ± 240.57	0.103
*NGLDM business*	0.021 ± 0.014	0.041 ± 0.048	**0.020**	0.021 ± 0.013	0.036 ± 0.037	**0.029**
*NGLDM complexity*	5397.69 ± 2467.22	4629.23 ± 1541.69	0.101	5112.13 ± 2020.06	4386.96 ± 1276.61	0.060
*NGLDM strength*	77.18 ± 32.79	75.43 ± 29.10	0.802	80.49 ± 37.66	79.68 ± 48.73	0.934

† Student’s *t*-test, mean ± standard deviation;

cisPCa, clinically insignificant prostate cancer; csPCa, clinically significant prostate cancer; ADC, apparent diffusion coefficient; GLCM, gray-level cooccurrence matrix; NGLDM, neighborhood gray-level different matrix.

**Table 3:** Univariate and multivariate logistic regression analyses for the texture parameters extracted from ADC maps and T2-weighted images in the peripheral zone.

	ADC maps				T2-weighted images			
	Univariate analysis		Multivariate analysis		Univariate analysis		Multivariate analysis	
	OR	p	OR	p	OR	p	OR	p
***First-order***								
*Mean*	0.988	**<0.001**	0.988	**0.002**	0.991	**<0.001**	0.993	**<0.001**
*Median*	0.989	**<0.001**	NA	NA	0.988	**<0.001**	NA	NA
*Skewness* †	1.267	**<0.001**	NA	NA	1.002	**0.002**	NA	NA
*Kurtosis* †	0.996	0.825	NA	NA	1.001	**0.026**	NA	NA
*Entropy* ‡	0.550	**<0.001**	0.621	**<0.013**	0.410	**<0.001**	0.473	**0.001**
*Uniformity* †	1.078	**0.009**	NA	NA	1.445	**<0.001**	NA	NA
***Second-higher-order***								
*GLCM contrast*	1.007	**0.014**	0.970	**0.040**	1.015	**0.006**	NA	NA
*GLCM correlation* †	0.998	0.085	NA	NA	0.993	**<0.001**	NA	NA
*GLCM difference entropy* ‡	0.920	0.132	NA	NA	1.080	0.300	NA	NA
*GLCM joint energy* †	1.072	**0.035**	1.224	0.080	1.580	**0.008**	NA	NA
*GLCM joint entropy* ‡	0.953	**0.015**	0.671	**0.031**	0.900	**0.003**	NA	NA
*GLCM invers variance* †	0.992	**0.029**	0.978	0.097	0.982	**0.014**	NA	NA
*GLCM sum entropy* ‡	0.924	**0.011**	2.252	**0.012**	0.618	**<0.001**	0.708	**0.015**
*NGLDM coarseness* †	1.004	0.616	NA	NA	1.021	0.104	NA	NA
*NGLDM contrast*	1.398	0.108	NA	NA	5.313	**0.020**	NA	NA
*NGLDM business* †	1.081	**<0.001**	1.448	**0.001**	1.051	**0.019**	1.093	**0.007**
*NGLDM complexity*	1.000	0.761	NA	NA	1.000	0.546	NA	NA
*NGLDM strength*	1.003	0.504	NA	NA	1.016	0.154	NA	NA

OR, odds ratio; CI, confidence interval; ADC, apparent diffusion coefficient; GLCM, gray-level cooccurrence matrix; NGLDM, neighborhood gray-level different matrix; NA, not available;

† OR in units of 1000;

‡ OR in units of 10.

**Table 4:** Univariate and multivariate logistic regression analyses for the texture parameters extracted from early and late T1-weighted images in the peripheral zone.

	Early T1-weighted images				Late T1-weighted images			
	Univariate analysis		Multivariate analysis		Univariate analysis		Multivariate analysis	
	OR	p	OR	p	OR	p	OR	p
***First-order***								
*Mean*	1.001	0.665	NA	NA	1.021	**<0.001**	NA	NA
*Median*	1.000	0.717	NA	NA	1.022	**<0.001**	1.044	**<0.001**
*Skewness* †	1.000	0.981	NA	NA	0.999	**0.043**	NA	NA
*Kurtosis* †	1.000	0.569	NA	NA	1.000	0.663	NA	NA
*Entropy* ‡	0.844	**0.002**	1.404	0.230	0.848	**0.002**	NA	NA
*Uniformity* †	1.068	**0.003**	1.202	0.083	1.060	**0.003**	1.152	**<0.001**
***Second-higher-order***								
*GLCM contrast*	1.004	**0.041**	NA	NA	1.005	**0.020**	NA	NA
*GLCM correlation* †	0.999	0.146	NA	NA	0.997	**0.009**	0.997	**0.042**
*GLCM difference entropy* ‡	0.763	**0.004**	NA	NA	0.784	**0.003**	0.767	**0.006**
*GLCM joint energy* †	1.080	**0.010**	1.923	**0.001**	1.081	**0.008**	NA	NA
*GLCM joint entropy* ‡	0.950	**0.015**	1.625	**0.001**	0.948	**0.011**	NA	NA
*GLCM invers variance* †	0.990	0.074	NA	NA	0.995	0.321	NA	NA
*GLCM sum entropy* ‡	0.914	**0.006**	NA	NA	0.900	**0.003**	NA	NA
*NGLDM coarseness* †	1.002	0.672	NA	NA	0.996	0.640	NA	NA
*NGLDM contrast*	1.205	0.052	NA	NA	1.180	**0.025**	NA	NA
*NGLDM business* †	1.034	**0.023**	NA	NA	1.027	**0.045**	NA	NA
*NGLDM complexity*	1.000	0.114	NA	NA	1.000	0.075	NA	NA
*NGLDM strength*	0.998	0.799	NA	NA	1.000	0.932	NA	NA

OR, odds ratio; CI, confidence interval; ADC, apparent diffusion coefficient; GLCM, gray-level cooccurrence matrix; NGLDM, neighborhood gray-level different matrix; NA, not available;

† OR in units of 1000;

‡ OR in units of 10.

**Table 5:** Receiver operating characteristic curve analysis results of multivariate regression models for clinically significant prostate cancer in peripheral zone.

	AUC †	Sensitivity (%)	Specificity (%)	p
*First-order logistic regression model based on ADC maps*	0.996 (0.989–1)	100	95.12	**<0.001**
*Second-higher-order logistic regression model based on ADC maps*	0.883 (0.814–0.952)	74.4	85.4	**<0.001**
*First-order logistic regression model based on T2WIs*	0.932 (0.873–0.992)	94.87	87.80	**< 0.001**
*Second-higher-order logistic regression model based on T2WIs*	0.891 (0.819–0.963)	84.6	85.4	**< 0.001**
*First-order logistic regression model based on early T1WIs*	0.729 (0.616–0.843)	51.3	92.7	**< 0.001**
*Second-higher-order logistic regression model based on early T1WIs*	0.771 (0.656–0.887)	69.2	95.1	**<0.001**
*First-order logistic regression model based on late T1WIs*	0.935 (0.886–0.984)	94.90	78.00	**<0.001**
*Second-higher-order logistic regression model based on late T1WIs*	0.759 (0.651–0.866)	53.85	90.24	**<0.001**

† AUC, area under curve (95% CI);

ADC, apparent diffusion coefficient; T2WIs, T2-weighted images; T1WIs, T2-weighted images
